# Neutrophil Elastase Degrades Histone Deacetylases and Sirtuin 1 in Primary Human Monocyte Derived Macrophages

**DOI:** 10.3390/ijms25084265

**Published:** 2024-04-12

**Authors:** Shuo Zheng, Gamze B. Bulut, Apparao B. Kummarapurugu, Jonathan Ma, Judith A. Voynow

**Affiliations:** Department of Pediatric Pulmonary Medicine, Children’s Hospital of Richmond at Virginia Commonwealth University, Richmond, VA 23219, USA; shuo.zheng@vcuhealth.org (S.Z.); gbbulut@wm.edu (G.B.B.); apparao.kummarapurugu@vcuhealth.org (A.B.K.); jonathan.ma@vcuhealth.org (J.M.)

**Keywords:** neutrophil elastase, macrophage, HDACs, Sirtuin, HMGB1, cystic fibrosis, COPD

## Abstract

Neutrophil elastase (NE) is taken up by macrophages, retains intracellular protease activity, and induces a pro-inflammatory phenotype. However, the mechanism of NE-induced pro-inflammatory polarization of macrophages is not well understood. We hypothesized that intracellular NE degrades histone deacetylases (HDAC) and Sirtuins, disrupting the balance of lysine acetylation and deacetylation and resulting in nuclear to cytoplasmic translocation of a major alarmin, High Mobility Group Box 1 (HMGB1), a pro-inflammatory response in macrophages. Human blood monocytes were obtained from healthy donors or from subjects with cystic fibrosis (CF) or chronic obstructive pulmonary disease (COPD). Monocytes were differentiated into blood monocyte derived macrophages (BMDMs) in vitro. Human BMDMs were exposed to NE or control vehicle, and the abundance of HDACs and Sirtuins was determined by Western blotting of total cell lysates or nuclear extracts or determined by ELISA. HDAC, Sirtuin, and Histone acetyltransferase (HAT) activities were measured. NE degraded most HDACs and Sirtuin (Sirt)1, resulting in decreased HDAC and sirtuin activities, with minimal change in HAT activity. We then evaluated whether the NE-induced loss of Sirt activity or loss of HDAC activities would alter the cellular localization of HMGB1. NE treatment or treatment with Trichostatin A (TSA), a global HDAC inhibitor, both increased HMGB1 translocation from the nucleus to the cytoplasm, consistent with HMGB1 activation. NE significantly degraded Class I and II HDAC family members and Sirt 1, which shifted BMDMs to a pro-inflammatory phenotype.

## 1. Introduction

Free neutrophil elastase (NE) is a major culprit for disease progression in both cystic fibrosis (CF) and chronic obstructive pulmonary disease (COPD). NE is abundant in the airways of patients with CF and COPD due to neutrophilic inflammation in response to chronic infections [1]. NE activates signaling pathways leading to airway remodeling, failure to control infections, and chronic inflammation. NE degrades innate immune functions of the sentinel airway cell, the macrophage, by several mechanisms [2]. NE causes macrophage phagocytic failure in part by cleaving opsonins, opsonin receptors, and macrophage receptors. NE sustains airway inflammation by stimulating NFκB upregulation of cytokines, degrading antimicrobial proteins such as lactoferrin, and activating other proteinases including cathepsins and matrix metalloproteases. NE treatment increases the release of TNFα, an M1 marker, from blood monocyte-derived macrophages (BMDMs). In contrast, NE does not increase CCL18, a M2 marker, consistent with NE-induced M1 macrophage polarization [3].

Recently, we reported that NE is taken up by human blood monocyte-derived macrophages (BMDMs), is localized to both the nucleus and cytoplasmic domains, and retains intracellular proteolytic activity [3]. NE clips histone H3 causing chromatin decondensation and triggering the release of macrophage extracellular traps in alveolar macrophages from Cftr-null or wild-type littermate mice [3] and from BMDMs from patients with CF [3] and from patients with COPD or control subjects [3,4]. Because NE cleaves peptides with alanine or valine at the P1 site [5], NE likely has many protease targets in the macrophage nucleus and cytosol. Therefore, we evaluated whether NE degrades histone post-translational modifying enzymes including Histone deacetylases (HDACs) and Sirtuins in BMDMs that could modify the macrophage phenotype.

HDACs and Sirtuins are evolutionarily conserved enzymes that remove the acetyl group from lysine with functional consequences such as chromatin condensation, transcriptional silencing, and signaling [6]. HDACs are classified based on their structure, function, and homology of accessory domains. There are 18 different human histone deacetylases (HDACs) including Class I nuclear HDACs (HDAC1-2-3-8), Class II cytoplasmic HDACs (HDACs4-5-6-7-9-10), Class III HDACs also known as Sirtuins (Sirt 1 through 7), and Class IV HDAC (HDAC11) [7].

Importantly, in COPD, changes in airway or serum HDAC and Sirt 1 concentrations have been reported. HDAC2 expression and HDAC activity are decreased in PBMCs in COPD patients compared with smokers and non-smokers [8]. Serum Sirt 1 levels are also decreased in patients with COPD [9]. Importantly, Sirt 1 is required to deacetylate High Mobility Group Box 1 (HMGB1), a chromatin binding protein, which normally resides in the nucleus. When Sirt 1 is degraded, HMGB1 is acetylated and translocates from the nucleus to the cytoplasm, a prerequisite for extracellular release and alarmin activity. Therefore, the Sirt 1-HMGB1 axis modulates lung inflammation [10]. We hypothesized that NE may alter the balance of histone deacetylases (HDACs or Sirtuins) and histone acetyltransferases (HATs) resulting in unopposed lysine acetylation of HMGB1 and cytoplasmic residence. To determine whether NE degrades specific HDACs or Sirts, we prepared human BMDMs from healthy individuals or from subjects with CF or COPD to assess the impacts of NE on HDAC and Sirt protein abundance, on deacetylase activity, and on HMGB1 cellular localization.

## 2. Results

**Figure 1:**
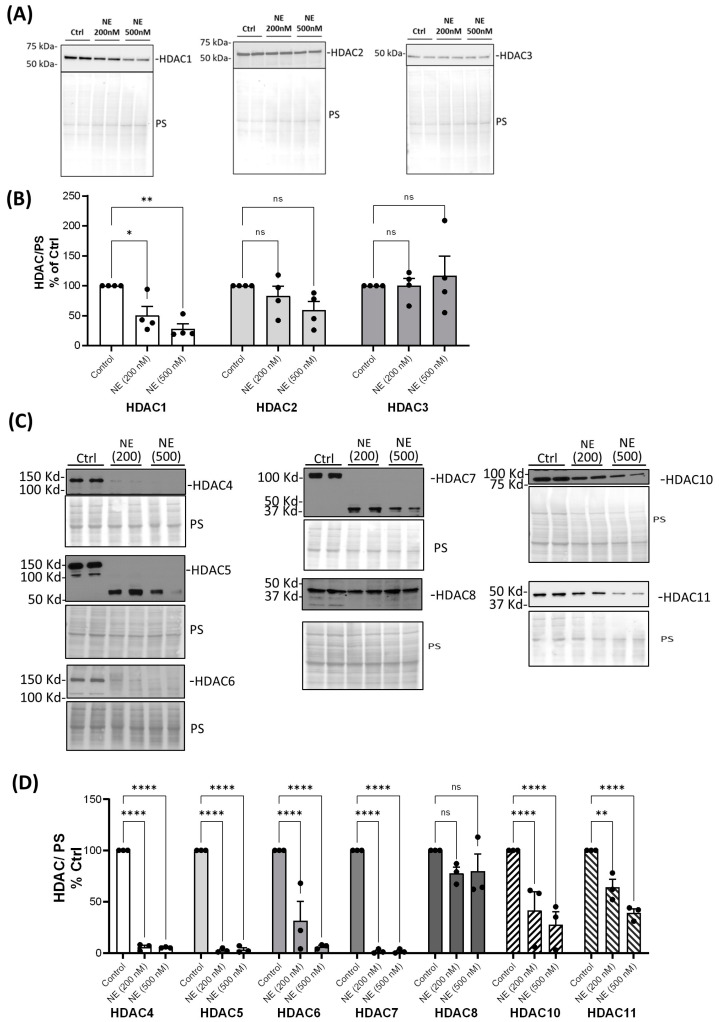
Neutrophil elastase degraded HDACs from healthy BMDM cell lysates. Human BMDMs were treated with either control vehicle (Ctrl) or NE (200 nM and 500 nM) for 2 h. (**A**) Nuclear extracts or (**C**) total cell lysates were separated using SDS-PAGE. Western blotting was performed using antibodies against HDAC1, HDAC2, HDAC3, HDAC4, HDAC5, HDAC6, HDAC7, HDAC8, HDAC10, and HDAC11. Antibodies and dilutions used for Western blot are listed in Table 1. The band intensities were quantified using Image J 1.52a (Wayne Rasband, NIH, USA). The relative HDAC expression was first normalized to total Ponceau S (PS) staining and then normalized to the control-treated cells. (**B**) HDAC1-3, *n* = 4 donors. (**D**) HDAC 4-11, *n* = 3 donors, with two replicates averaged per treatment condition. Data (mean ± SEM) were analyzed by Prism using one-way, nonparametric ANOVA (Kruskal–Wallis test), followed by Dunn’s multiple comparison. *, *p* < 0.05; **, *p* < 0.01; ****, *p* < 0.0001; ns, not significant.

**Figure 2:**
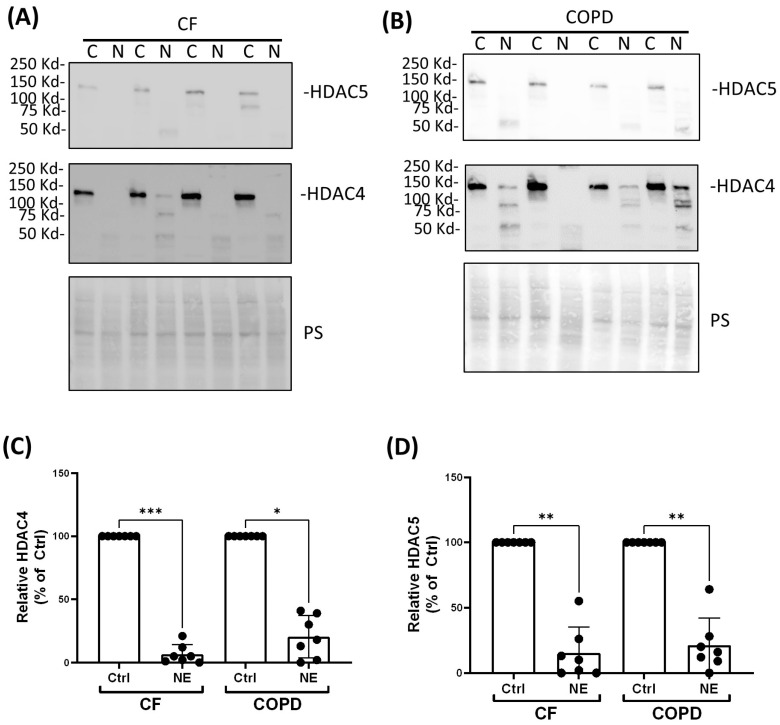
Neutrophil elastase degraded HDAC4 and HDAC5 in BMDMs from patients with CF and COPD. Human BMDMs from CF (**A**) or COPD (**B**) were treated with either control vehicle (Ctrl) or NE (200 nM) for 2 h. Total cell lysates were separated using SDS-PAGE. Western blotting was performed using antibodies against HDAC4 and HDAC5. (**C**,**D**) Band intensities were quantified using Image J. Relative HDAC4 or HDAC5 expression was first normalized to total Ponceau S (PS) staining and then normalized to control-treated cells; *n* = 7 donors. Data (mean ± SEM) were analyzed by Prism using one-way, nonparametric ANOVA (Kruskal–Wallis test), followed by Dunn’s multiple comparison. *, *p* < 0.05; **, *p* < 0.01; ***, *p* < 0.001.

**Figure 3:**
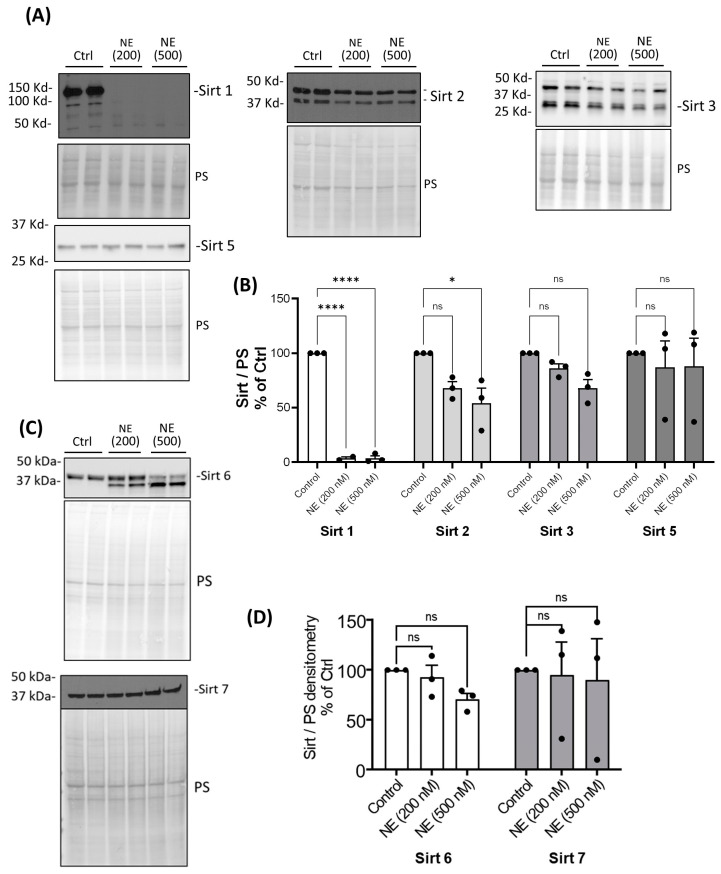
Neutrophil elastase degraded Sirt 1 in healthy BMDM cell lysates. Human BMDMs were cultured and treated with either control vehicle or NE (200 nM and 500 nM) for 2 h. (**A**) Total cell lysates or (**C**) nuclear extracts were separated using SDS-PAGE. Western blotting was performed using antibodies against Sirt 1, Sirt2, Sirt3, Sirt 5, Sirt 6, and Sirt 7. (**B**,**D**) Band intensities were quantified using Image J. Relative Sirt expression was first normalized to total Ponceau S (PS) staining and then normalized to the control-treated cells; *n* = 3 donors with two replicates averaged per treatment condition. Data (mean ± SEM) were analyzed by Prism using one-way, nonparametric ANOVA (Kruskal–Wallis test), followed by Dunn’s multiple comparison. *, *p* < 0.05; ****, *p* < 0.0001; ns, not significant.

**Figure 4:**
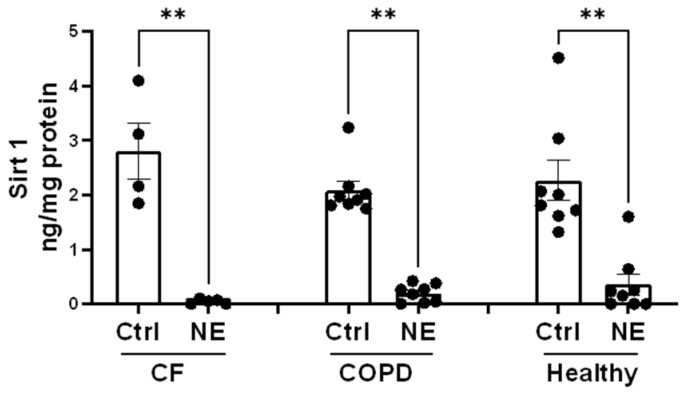
Neutrophil elastase decreased Sirt 1 in total cell lysates from patients with CF and COPD and healthy controls. Total cell lysate from Ctrl or NE (200 nM, 2 h) treated hBMDMs from Buffy coat (healthy) and patients with CF or COPD were evaluated for Sirt 1 protein by ELISA (Abcam, Ab171573). The Sirt 1 protein level was normalized to the total protein level (ng/mg protein) and presented as the mean ± SEM. Heathy, *n* = 8; CF, *n* = 4; COPD, *n* = 8. Statistical analysis was performed by Prism using one-way, nonparametric ANOVA (Kruskal–Wallis test), followed by Dunn’s multiple comparisons test, **, *p* < 0.01.

We examined whether NE catalyzed the degradation of HDAC and Sirtuin enzymes and decreased their activity. There are 18 different human histone deacetylases (HDACs). Some of them are located in the nucleus, and some of them are located in both the cytoplasm and the nucleus. We first screened most of the HDACs and Sirtuins by Western analysis using either nuclear extracts or total cell lysates of BMDMs from healthy buffy coat donors. NE (200 nM and 500 nM, 2 h) caused a significant decrease in nuclear HDAC 1 but had no effect on the protein abundance of other Class I nuclear HDACs: HDAC2 and HDAC3 (Figure 1A,B). NE (200 nM and 500 nM, 2 h) caused a significant decrease in cytoplasmic Class II and IV HDAC4, 5, 6, 7, 10, and 11 but had no effect on HDAC8 (Figure 1C,D). To confirm the finding in BMDMs from patients with CF and COPD, we chose to use one dose of NE (200 nM, 2 h) and total cell lysate for Western analysis. Similarly, NE (200 nM, 2 h) decreased HDAC4 and HDAC5 in BMDMs from patients with CF and COPD (Figure 2). NE (200 nM and 500 nM, 2 h) decreased Sirtuin 1 but had no significant effect on Sirtuins 3 or 5. Sirtuin 2 was decreased by NE at 500 nM (Figure 3A,B). Sirtuin 6 was clipped by NE (200 nM and 500 nM, 2 h), resulting in double bands in NE-treated samples. Quantitation of the top band, which corresponded to the full-length Sirtuin 6, showed a decrease in expression compared with the control-treated sample, but the decrease was not statistically significant. There was no significant NE effect on Sirtuin 7 (Figure 3C,D). Importantly, NE (200 nM, 2 h) decreased Sirt 1 expression in total cell lysates of BMDMs from patients with CF and COPD by ELISA (Figure 4).

**Figure 5:**
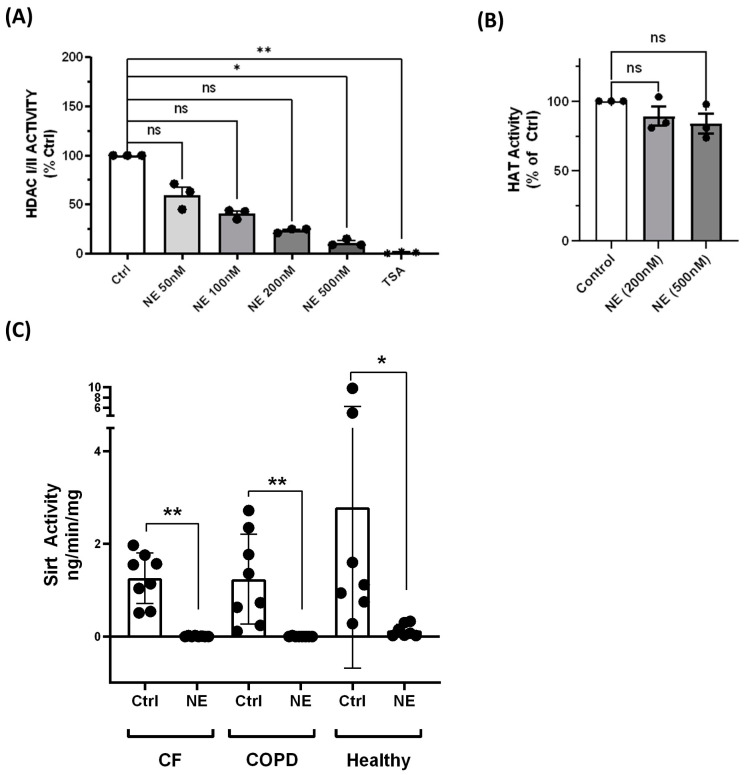
Neutrophil elastase decreased HDAC and Sirtuin activity but not HAT activity. (**A**) BMDMs from healthy buffy coat donors seeded in 96-well plates were treated with either control vehicle (Ctrl), NE (50 nM–500 nM), or TSA (1 µM), for 2 h. HDAC I/II activity was determined using a Promega HDAC-Glo I/II assay kit. The results were normalized to Ctrl-treated samples and expressed as the relative HDAC activity (mean ± SEM). *n* = 3 individuals with triplicate samples per donor. Statistical analysis was performed by Prism using one-way, nonparametric ANOVA (Kruskal–Wallis test), followed by Dunn’s multiple comparisons test. *, *p* < 0.05; **, *p* < 0.01; ns, not significant. (**B**) Human BMDMs from healthy buffy coat donors were treated with either control vehicle or NE (200 nM and 500 nM) for 2 h. Nuclear protein was used for HAT activity using HAT activity kits. Relative HAT activity was normalized to control treated samples; *n* = 3 donors with two replicates averaged per treatment condition. Data (mean ± SEM) were analyzed by Prism using one-way, nonparametric ANOVA (Kruskal–Wallis test), followed by Dunn’s multiple comparison. ns, not significant. (**C**) Total cell lysate from Ctrl or NE (200 nM, 2 h) treated hBMDMs from Buffy coat (healthy) and patients with CF or COPD were evaluated for Sirt activity (Abcam, Ab1156915). Sirt activity was expressed as ng/min/mg total protein and presented by mean ± SEM. Heathy, *n* = 7; CF, *n* = 8; COPD, *n* = 8. The Wilcoxon matched-pairs signed rank test was performed (Prism) between the control and the NE-treated samples in each group. **, *p* < 0.01; *, *p* < 0.05.

To determine whether NE affected HDAC activity, we seeded BMDMs (grown in suspension) in 96-well plates and treated them with either control vehicle (Ctrl), NE (50 nM–500 nM), or TSA (1 µM), for 2 h, followed by an in-cell HDAC-Glo I/II assay. HDAC-Glo I/II assay kits are able to detect HDAC activity of Class I nuclear HDACs (HDAC1-2-3-8), Class II cytoplasmic HDACs (HDAC4-5-6-7-9-10), and a Class IV HDAC (HDAC11). The NE treatment (50 nM–500 nM, 2 h) resulted in a concentration-dependent loss of total HDAC activity (Figure 5A), consistent with the NE-induced decrease in HDAC expression. The two major enzyme classes that modify protein acetylation include histone acetyltransferases (HATs), which catalyze lysine acetylation, and HDACs and Sirtuins, which catalyze lysine deacetylation. Changes in abundance or activity alter the equilibrium of histone/protein acetylation. To determine whether NE affects HAT activity, we measured HAT activity using nuclear extracts of BMDMs from healthy subjects. NE (200 nM and 500 nM, 2 h) did not affect the total HAT activity (Figure 5B). Since Sirt 1 is the major Sirtuin regulated by NE treatment, we used a universal Sirt activity assay kit to determine the effect of NE treatment on Sirt 1 activity, using total cell lysate. The NE (200 nM, 2 h) treatment decreased the total Sirt activity in BMDMs from healthy subjects and from patients with CF and COPD (Figure 5C), consistent with the NE-induced decrease in Sirt 1 expression.

**Figure 6:**
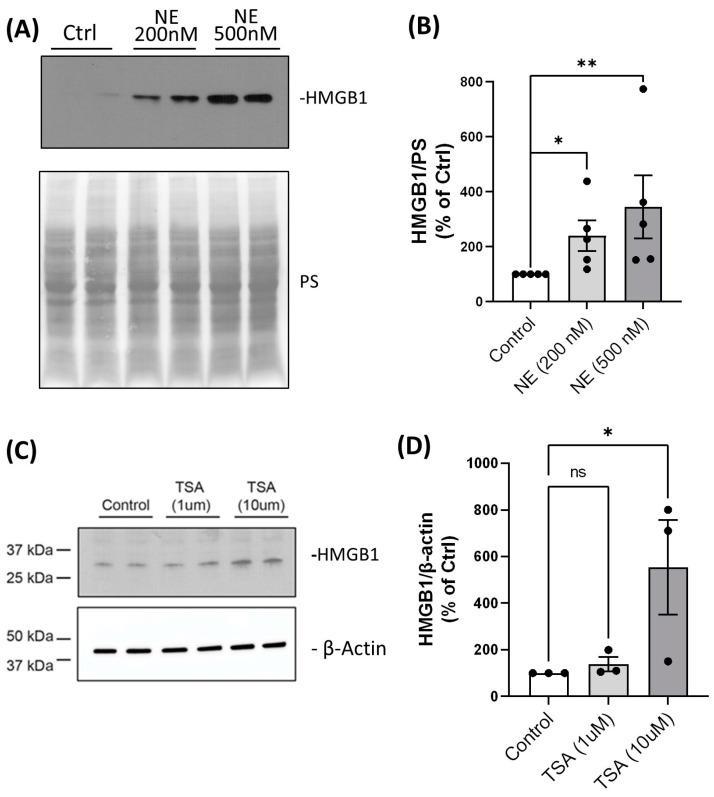
NE and TSA treatment increased HMGB1 abundance in cytoplasm. (**A**) BMDMs from healthy buffy coat donors were treated with either control vehicle or NE (200 nM and 500 nM) for 2 h. Cytoplasmic extracts used for Western blotting of HMGB1. (**B**) HMGB1 band intensities were quantified using Image J, normalized to total Ponceau S (PS) staining, and expressed as a percentage of control. Data were summarized from *n* = 5 donors with two replicates per treatment condition and expressed as mean ± SEM. (**C**) BMDMs from healthy buffy coat donors were treated with either control vehicle or TSA (1 µM and 10 µM) for 24 h, and cytoplasmic extracts were used for Western analysis of HMGB1 and β-actin. (**D**) Band intensities were quantified using Image J. HMGB1 was normalized to β-actin and expressed as a percentage of control. Data were summarized from *n* = 3 donors with two replicates per treatment condition. Data (mean ± SEM) were analyzed by Prism using one-way, nonparametric ANOVA (Kruskal–Wallis test), followed by Dunn’s multiple comparison. ns, not significant. *, *p* < 0.05; **, *p* < 0.01; ns, not significant.

When HAT/HDAC equilibrium is perturbed, increased acetylation of HMGB1 at several key lysine residues will determine its cellular localization, causing a shift from the nucleus to the cytoplasm. We tested whether the loss of HDAC/Sirtuin activity resulted in HMGB1 translocation from the nucleus to the cytoplasm by Western analysis. We demonstrated that there was an NE-induced concentration-dependent increase in HMGB1 abundance in the cytoplasm (Figure 6A,B). BMDMs were treated with TSA (1 µM and 10 µM, 24 h), which also increased the cytoplasmic abundance of HMGB1 in a concentration-dependent manner (Figure 6C,D). These experiments are consistent with HDAC loss or inhibition and unopposed lysine acetylation, driving nuclear to cytoplasmic translocation of HMGB1, a prerequisite condition for extracellular release of HMGB1.

## 3. Discussion

*NE and HDAC degradation*: Herein, we demonstrated that NE proteolytically degraded HDAC1 (Class I); HDAC4, 5, 6, 7, and 10 (Class II); HDAC11 (Class IV); and Sirt 1 (Class III), resulting in a concentration-dependent loss of HDAC activity. The NE-induced loss of HDAC activity was specific because in contrast, there was no significant change in HAT activity. Furthermore, treatment with the HDAC inhibitor, TSA, confirmed that HMGB1 nuclear to cytoplasm translocation was associated with a loss of HDAC activity. We previously reported that the inhibition of p300 HAT activity by 2-O, 3-O desulfated heparin was sufficient to block NE- or LPS-induced HMGB1 release from a murine macrophage cell line, RAW264.7 [11]. Thus, the balance of HAT and HDAC activities control HMGB1 lysine acetylation, cellular localization, and function.

*HDAC and HMGB1*: Previous publications demonstrate the relationship between the loss of HDAC activity and HMGB1 post-translational modifications and cellular fate. Ischemia and reperfusion injury, associated with the loss of HDAC 1 and 4 [12], or cerebral ischemia and reperfusion injury associated with the loss of HDAC 4 and 5 [13] were both associated with HMGB1 translocation or cellular release. In murine renal endothelial cells, following ischemia and reperfusion injury, Sirt 1 inhibition is associated with lysine acetylation and cytoplasmic translocation of HMGB1; this process is rescued by the introduction of recombinant Sirt 1 [14]. In HEK293T cells, the lysine-acetylation status of the HMGB1 Nuclear Localization Site 1 (NLS1) regulates the interaction with Sirt 1. When HMGB1 is hypoacetylated, it interacts with Sirt 1 and is sequestered in the nucleus. However, with LPS or TNFα exposure, HMGB1 nuclear localization signal 1 (NLS1) lysine residues are acetylated, shifting HMGB1 to interact with the nuclear exporter, chromosome region maintenance 1 (CRM1), and cytoplasmic localization [15]. Thus, acetylation of several key HMGB1 lysine residues controls cell localization and functional status.

*Cytoplasmic accumulation of HMGB1*: Cytoplasmic accumulation of HMGB1 in secretory lysosomes is a pathogenic feature of COPD, cigarette smoke extract (CSE), and ventilator-induced lung injury [16,17,18] and is a prerequisite for extracellular HMGB1 release. In a murine model of ventilator-induced lung injury, increased cytoplasmic accumulation of HMGB1 is associated with JAK2/STAT1 activation, autophagy, apoptosis, and LDH release [18]. The treatment of macrophages with extracellular rhHMGB1 did not activate autophagy, suggesting that endogenous HMGB1 was sufficient for CSE-induced macrophage inflammation [17].

*HDACs and COPD and CF*: Oxidative stress is associated with a loss of HDACs in COPD and CF. HDAC2, 5, and 8 mRNA are downregulated in the lung tissue of patients with COPD, and both HDAC2 protein and activity are decreased in COPD lung macrophages [8]. CSE decreases HDAC class I activity in macrophages [19] via decreased glutathione and increased nitration modification of HDACs. CSE decreases HDAC3 in human BMDMs resulting in IL8 and IL1β upregulation in conditioned media [20]. Sirt 1 protein and activity are markedly reduced in the blood monocytes of patients with COPD, regardless of smoking status [21]. HDAC2 protein and activity levels are decreased in CF human primary nasal cells and in CF human epithelial cell lines via a post-transcriptional oxidative mechanism. The loss of HDAC2 is associated with upregulation of IL-8 expression with associated H4-chromatin acetylation at the IL-8 promoter domain [22].

*Proteases and HDAC regulation:* To our knowledge, our report is the first to identify that an exogenous human protease, NE, was sufficient to decrease HDAC protein levels and activity in BMDMs. Importantly, the NE concentrations used were relevant to concentrations of NE reported in sputum from patients with COPD [23] or CF [24].

In addition to NE, there are several reports of viral proteinases or viral-regulated host caspases that degrade HDACs resulting in altered innate immune function. SARS-CoV-2 and Porcine deltacoronavirus proteinase, Nsp5, cleave class 1 HDACs resulting in a loss of HDAC activity and the failure of interferon-stimulated gene expression, an anti-viral immune mechanism [25,26]. Lysosomal caspase 3, activated by Influenza A, degrades HDAC6 [27], resulting in increased viral protein transcription. Lysosomal caspase 3 also degrades HDAC4, resulting in diminished innate immune function [28]. Under conditions of cellular stress or transformation, activated caspase 3 cleaves HDACs and Sirt 1 during apoptosis [29,30,31], autophagy, and senescence [32,33]. Thus, proteases regulate HDAC expression and function. Importantly, we demonstrate that NE plays a critical role in the balance of HDACs and HATs resulting in the activation of HMGB1 in BMDMs.

## 4. Materials and Methods

### 4.1. Human Subjects

Buffy coats from deidentified healthy donors were obtained from the American Red Cross (Washington, DC, USA). All subjects were Caucasian except 1 subject who identified as mixed race, and the subjects included 9 females and 10 males and an age range of 25–89 years. Subjects with COPD and subjects with CF provided written informed consent approved by the VCU IRB (HM20015308 and HM20018160, respectively). Subjects with COPD included 5 African Americans and 9 Caucasians, 5 females and 9 males, 6 current smokers and 8 past smokers, 8 subjects assigned Gold stage A/B and 6 subjects assigned Gold stage D, and an age range from 55 to 79 years. Subjects with CF were all Caucasian except for 1 African American subject and included 8 females and 4 males, with 10 subjects on Trikafta, 1 subject on Ivacaftor, and 1 subject on no highly effective CFTR modulator therapy. The ages of subjects with CF ranged from 7 to 60 years.

### 4.2. Buffy Coat Processing Using Double Density Gradients for Human Peripheral Blood Monocyte-Derived Macrophage (BMDM) Cultures

Buffy coats were diluted in RPMI 1640 medium (1:3) and layered onto Lymphopure (15 mL, Biolegend, #426202, San Diego, CA, USA) in a 50 mL conical tube. After centrifugation at 1000× *g*, 15 min with the brake off, the intermediate cloudy layer was collected, washed, and incubated with red blood cell lysis buffer to obtain peripheral blood mononuclear cells (PBMCs). PBMCs (50–70 M cells/mL; up to 200 M cells) were layered onto a second gradient (10 mL of Percoll gradient solution: 23.5 mL Percoll (GE Healthcare Life Sciences, Marlborough, MA, USA), 5 mL 1.6 M NaCl, 21.5 mL H_2_O at 1.0661 density [34]) in a 15 mL conical tube and centrifuged at 600× *g* for 15 min with the brake off. The intermediate cloudy monocyte layer was collected, washed, and counted. Monocytes were seeded on 10 cm tissue culture plates (37 °C, 1 h). Unattached cells were removed by aspiration. Attached monocytes were cultured in RPMI growth media (500 mL RPMI 1640 medium (Quality Biological, Gaithersburg, MD, USA), 10% fetal bovine serum (FBS; Life technologies, Carlsbad, CA, USA), 1:100 L-Glutamine, 1:100 Pen/Strep, 1 M HEPES (1.25 mL)) and GM-CSF (20 ng/mL, Biolegend, Cat# 572903) for 8–10 days, with a media change every other day. BMDMs isolated by double density gradients and differentiated by GM-CSF in RPMI media were used for treatment and to collect total cell lysate or cytoplasmic and nuclear extracts for HDAC and Sirtuin Western analyses and HAT activity assay. We confirmed the purity of the BMDM by flow cytometry using CD11b and CD68 and by cell morphology (cytospin followed by Dif-Quick staining of the cells). GM-CSF differentiation generates BMDMs with cell surface receptors expressed by alveolar macrophages (AMs) [35].

### 4.3. Monocyte Enrichment via Rosette-Sep Using Buffy Coat and Patient Whole Blood

Whole blood samples from patients with CF and COPD or buffy coat samples (diluted 1:2 with PBS dilution buffer (PBS+ 2% FBS+ 1 mM EDTA)) were processed using a Rosette-Sep human monocyte enrichment cocktail (Stem Cell Technologies, #15068, Seattle, WA, USA) following the manufacturer’s instructions. Monocytes were cultured in suspension for 7 days in RPMI growth medium with GM-CSF (20 ng/mL) to differentiate to macrophages. We confirmed the purity of the BMDMs using CD11b and CD68. Macrophages from the buffy coat were seeded on a 96-well plate (white wall, transparent bottom) for additional 2–3 days for HDAC activity assay. Macrophages from patients with CF or COPD were seeded in 12-well plate for NE treatment and total cell lysate collection.

### 4.4. Treatments of BMDMs with NE and Trichostatin A (TSA); Total Cell Lysate Collection and Cell Fractionation

BMDMs were stimulated with NE (Elastin Products, SE563) in serum-free media for 2 h. At the end of the NE treatment, an NE-specific inhibitor, N-(Methoxysuccinyl)-Ala-Ala-Pro-Val-chloromethyl ketone (AAPV-CMK, Sigma, St. Louis, MO, USA, M0398, final concentration 10 µM), was added to stop NE activity. Total cell lysates were prepared in a lysis buffer (CST #9803) containing protease and phosphatase inhibitors (Sigma, P8340, P5726) for HDAC/Sirtuin Westerns. Nuclear and cytoplasmic extracts were prepared using a nuclear extract kit (Active Motif, # 40010, Carlsbad, CA, USA) following the manufacturer’s instructions, except that DTT was omitted for enzyme activity assays. BMDMs were treated with TSA, a global HDAC inhibitor (Sigma, T1952), in media with serum for 24 h to collect cytoplasmic extracts for HMGB1 Westerns [36].

### 4.5. Western Blotting and Antibodies

The total cell lysate, nuclear extracts, or cytoplasmic extracts (25 µg) were separated by 4–20% SDS PAGE (BioRad, 4561094) and transferred to a nitrocellulose membrane (BioRad, #1620112, Hercules, CA, USA). The membrane was stained with Ponceau S (Sigma, P7170), and the image was scanned. The membrane was then washed and used for Western analysis. Antibodies and dilutions are listed in Table 1. Westerns were developed by SuperSignal™ West Pico PLUS Chemiluminescent Substrate (ThermoFisher, #34579, Waltham, MA, USA) and band density determined by Image J (NIH). The band density was normalized to Ponceau S staining for each lane, a measure of the total protein quantitation. The normalized data from NE-treated cells were then compared to control vehicle-treated cells from the same individual and expressed as a percentage of control.

### 4.6. HDAC Activity Assays

The macrophages seeded on the 96-well plate were treated with NE (50–500 nM), TSA (1 µM), or control vehicle for 2 h. At the end treatment, AAPV-CMK was added to stop NE activity; cells were incubated with HDAC-Glo kit reagent mixture per the manufacturer’s instructions (Promega G6420 HDAC-Glo I/II assay, Madison, WI, USA), and luminescence was read using the SPARK plate reader (TECAN, Morrisville, NC, USA).

### 4.7. HAT Activity Assay

Nuclear extract (45 µg) was used for HAT activity using a HAT Assay kit (BioVision, K334-100, Milpitas, CA, USA) per the manufacturer’s instructions, using the SPARK plate reader.

### 4.8. Sirt 1 ELISA

The TCL from Ctrl or NE (200 nM, 2 h) treated hBMDMs from Buffy coat (healthy) or patients with CF or COPD was first diluted 5 times in cell extraction buffer and then evaluated for Sirt 1 protein by ELISA (Abcam, Ab171573, Waltham, MA, USA). The Sirt 1 protein level was normalized to the total protein in each reaction, and the results are presented as ng/mg protein.

### 4.9. Sirt Activity Assay

The total cell lysate from Ctrl or NE (200 nM, 2 h) treated hBMDMs from Buffy coat (healthy) and patients with CF or COPD was evaluated for Sirt activity (Abcam, Ab156915, Waltham, MA, USA). A quantity of 4 µL of sample was used for each reaction, and the Sirt activity was expressed as ng/min/mg total protein.

## 5. Conclusions

NE has myriad effects on cell processes relevant to chronic obstructive lung diseases (reviewed in [1,37]). We now demonstrate that NE decreased HDACs and Sirt 1 in BMDMs, activating HMGB1 nuclear to cytoplasmic translocalization. NE reduction in HDAC and Sirt activity likely has an even broader impact on the epigenetic regulation of macrophage gene expression and function.

## Figures and Tables

**Table 1:** Antibodies and dilutions used for Western analysis.

Target	Antibody (Dilution)	Target	Antibody (Dilution)
HDAC1	sc-81598 (1:1000)	HDAC10	sc-393417 (1:500)
HDAC2	CST #5113s (1:1000)	HDAC11	sc-390737 (1:500)
HDAC3	CST #3949s (1:1000)	Sirt 1	CST 9475T (1:1000)
HDAC4	sc-46672 (1:500)	Sirt 2	CST 12650 (1:1000)
HDAC5	sc-133106 (1:500)	Sirt 3	CST 5490 (1:1000)
HDAC6	CST #7558s (1:2000)	Sirt 5	CST 8782 (1:1000)
HDAC7	sc-74563 (1:500)	Sirt 6	CST 12486 (1:1000)
HDAC8	sc-374180 (1:500)	Sirt 7	CST 5360 (1:1000)
Β-actin	Sigma A5441 (1:8000)	HMGB1	sc-56698 (1:1000)
Mouse IgG, HRP	NXA931 (1:10,000)	rabbit IgG, HRP	CST 7074 (1:2000)

sc: Santa Cruz Biotechnology, Dallas, TX, USA; CST: Cell Signaling Technology, Danvers, MA, USA; NXA: GE Healthcare Life Sciences, Marlborough, MA, USA; Sigma: St. Louis, MO, USA.

## Data Availability

The raw data supporting the conclusions of this article will be made available by the authors on request.
